# Evaluation of the awareness of pediatric residents of anaphylaxis diagnosis through clinical scenarios

**DOI:** 10.1016/j.waojou.2025.101076

**Published:** 2025-06-07

**Authors:** Tülay Tuğçe Kutsal Gültekin, Ayşe Gökçe Kutsal, Gökhan Yörüsün, Ahmet Selmanoğlu, Kaan Çelebier, Zeynep Şengül Emeksiz, Emine Dibek Mısırlıoğlu

**Affiliations:** a University of Health Sciences, Ankara City Hospital, Department of Pediatric Allergy/Immunology, Ankara, Turkey; b University of Health Sciences, Ankara City Hospital, Department of Pediatrics, Ankara, Turkey

**Keywords:** Anaphylaxis, Adrenaline auto-injector, Pediatric

## Abstract

**Introduction:**

Anaphylaxis is an acute onset, life-threatening systemic hypersensitivity reaction requiring urgent medical intervention. Early recognition and appropriate treatment of anaphylaxis are crucial for patient survival. This study aims to assess the awareness of pediatric residents regarding the diagnostic criteria, differential diagnosis, and appropriate treatment approaches for anaphylaxis.

**Materials and methods:**

Active pediatric residents volunteering in participating in the study were included. Participants were given a questionnaire consisting of 10 clinical scenarios assessing diagnostic criteria and 15 questions evaluating professional experience and knowledge.

**Results:**

A total of 198 pediatric residents were included in the study. The average clinical working experience of the participants was 18 months. On average, participants answered 7.3 out of 10 clinical scenario questions correctly (min-max: 3–10). In the clinical scenario describing a 2-year-old patient with a history of anaphylaxis to milk-based formula, the patient developed coughing and wheezing after entering the kitchen while the mother was boiling milk. The patient had a respiratory rate of 52/min, SpO_2_ of 90%, and bilateral wheezing. This case, which involved no ingestion and presented only with signs of bronchospasm, was the scenario that participants found most challenging. While 64.1% of the participants classified it as anaphylaxis, 35.9% identified that the clinical presentation did not meet the diagnostic criteria for anaphylaxis. Similarly, the scenario involving a 17-year-old patient with acute rheumatic fever who developed syncope 5 min after receiving a benzathine penicillin injection and presented with confusion, heart rate of 58 bpm, blood pressure of 80/50 mmHg, respiratory rate of 38 breaths/min, SpO2 of 95%, and sinus bradycardia on ECG, was the least correctly identified as vasovagal syncope in terms of a differential diagnosis of anaphylaxis (33.8%). The results indicated that pediatric residents were less successful in diagnosing anaphylaxis in cases without skin/mucosal involvement and in drug/venom-related anaphylaxis cases. When both scenario-based and knowledge-based questions were considered, residents in their final 2 years and those who had received specialist training demonstrated significantly higher overall correct response rates (p < 0.001, p = 0.002). Epinephrine was selected as the first-line treatment in 99.5% of cases; 81.3% of participants correctly identified the dosage, 97.5% the route of administration, and 89.4% the site of administration.

**Conclusion:**

In our country, the emergency treatment and follow-up of pediatric patients experiencing anaphylaxis are mostly carried out by pediatric specialists. Therefore, the education and training on the diagnosis and emergency management of anaphylaxis, a pediatric emergency, hold significant importance during the pediatric residency training period.

## Introduction

Anaphylaxis is a life-threatening systemic allergic reaction characterized by acute onset symptoms that require immediate medical attention. Rapid recognition of anaphylaxis and prompt application of appropriate treatment methods are critical. Therefore, it is imperative that healthcare professionals constantly update their knowledge and skills regarding anaphylaxis in order to minimize morbidity and mortality rates.^1^

Several recent advancements have been reported in the diagnosis and management of anaphylaxis, including updates to diagnostic criteria by the World Allergy Organization in 2020 (WAO), increased availability and use of adrenaline auto-injectors, and enhanced educational efforts targeting recognition of atypical anaphylaxis presentations lacking cutaneous symptoms.^2^^,^^3^ However, little is known about whether all pediatricians are familiar with these developments. The rising incidence of anaphylaxis may be attributed to both the overall increase in the prevalence of allergic conditions and heightened awareness among pediatricians, and to specific training about the recognition and treatment of anaphylaxis.^4^

The National Institute of Allergy and Infectious Diseases (NIAID) and the Food Allergy and Anaphylaxis Network (FAAN) met in April 2004 to establish consensus and resolve disagreements on the diagnosis and management of anaphylaxis and established widely used criteria for anaphylaxis, which were updated in 2005. The 2011 WAO Criteria and the 2014 European Academy of Allergy and Clinical Immunology (EAACI) Criteria were the same as the 2005 symposium.^5^ However, in its 2020 update, WAO made a major change and accepted only laryngospasm or bronchospasm without typical skin manifestations as a finding of anaphylaxis as a result of “known or highly probable allergen exposure” in Article 2.^3^ The EAACI 2021 update remains unchanged.^1^ Considering that the diagnosis of anaphylaxis is made by clinical findings, it is crucial to apply the commonly accepted diagnostic criteria in the current practical approach.

The aim of this study was to assess pediatric residents' familiarity with current anaphylaxis guidelines, their perspectives on recognizing clinical scenarios indicative of anaphylaxis, and their competence in administering appropriate treatment.

## Methods

The current study was conducted at the Pediatric Allergy and Immunology Clinic of Ankara Bilkent City Hospital. Ethical approval for the study was obtained from the Clinical Research Ethics Committee (Decision No: TABED 2-24-111).

The study included pediatric residents who were actively working and volunteered to participate in the study. The residents' demographic characteristics such as age and gender were recorded, as well as their active working experience in pediatrics, pediatric allergy and immunology, and pediatric emergency department clinics. Additionally, the study explored their prior training on anaphylaxis and assessed whether they perceived their level of knowledge to be sufficient. Furthermore, an evaluation questionnaire was administered, consisting of 10 case scenarios addressing anaphylaxis diagnostic criteria and differential diagnosis. The questionnaire also included questions on professional experience, assessing basic information on anaphylaxis treatment, such as adrenaline application site, appropriate adrenaline dose, dosage information of commercially available adrenaline autoinjector preparations, and anaphylaxis treatment knowledge questions. Informed consent was obtained from all participants before administering the study questionnaire.

Statistical analysis of the data was performed using the SPSS v.22 statistical software package developed by IBM. Numbers and percentages were reported for discrete variables, while continuous variables were expressed using the mean and standard deviation if they were normally distributed, and median and interquartile range (IQR) for data that were not normally distributed.

## Results

The study population consisted of 198 pediatric residents who were actively receiving training in a pediatric clinic. Their median age was calculated as 28.8 years (IQR: 27–31). There were 160 (80.8%) female participants. Approximately 27.3% of them were in the first year of the training process. Thirty-three (16.7%) of the residents reported that they had never encountered a case of anaphylaxis during their rotations in the inpatient and outpatient clinics, or emergency department. 89 (44.9%) residents had received anaphylaxis training from an allergist, 62 (31.3%) from a pediatric specialist, and 28 (14.1%) from a senior resident.

The majority of participants expressed confidence in their knowledge of anaphylaxis diagnostic criteria, with 142 residents (71.7%) indicating that they were familiar with the current guidelines. Upon evaluating the participants' responses to the scenarios aimed at assessing their awareness of anaphylaxis diagnostic criteria, it was observed that they correctly answered an average of 7.3 out of 10 case-based questions (minimum: 3, maximum: 10, IQR: 6–8, median: 7). Five participants (2.5%) answered all case questions correctly. Data on case scenarios and correct answer rates are shown in Table 1. No significant difference was observed in the correct response rates to scenario-based questions among residents in the first or last 2 years of their training, or those who had or had not previously received anaphylaxis training from pediatric allergy specialists (p = 0.192, p = 0.354).

**Table 1:** Case scenarios and correct answer rates

Case scenario	Correct answer (n,%)
1) A 9-month-old female patient was brought to the pediatric emergency clinic 20 min after eating a boiled egg for breakfast, with symptoms of widespread skin redness, swelling of the lips, and audible wheezing from the chest. On physical examination, her general condition was moderate, and she was conscious. Her vital signs were as follows: Temperature: 37°C, heart rate: 136 bpm, blood pressure: 90/60 mmHg, respiratory rate: 56 breaths per minute, and oxygen saturation: 88%. The examination revealed urticarial rash all over the body, edema of the upper and lower lips, and inspiratory stridor on auscultation. - **Anaphylaxis-**	192 (97%)
2) A 10-year-old male patient presented to the emergency department with complaints of cramp-like abdominal pain, vomiting, and coughing, which developed within 5 min after taking the prescribed dose of Cefdinir on the first dose of treatment for tonsillitis. On physical examination, his general condition was moderate, and he was conscious. His vital signs were as follows: Temperature: 38.5°C, heart rate: 128 bpm, blood pressure: 110/70 mmHg, respiratory rate: 22 breaths per minute, and oxygen saturation: 92%. Respiratory sounds were coarse, with prolonged expiration. No rash was observed. - **Anaphylaxis-**	131 (66.2%)
3) A 16-year-old female patient with a known history of anaphylaxis to almonds was brought to the emergency department after losing consciousness 15 min after eating vegan ice cream. It was later learned that the ice cream was made with almond milk. On physical examination, her general condition was poor, and she was unconscious. Her vital signs were as follows: Temperature: 36.6°C, heart rate: 142 bpm, blood pressure: 75/48 mmHg, respiratory rate: 16 breaths per minute, and oxygen saturation: 95%. Her breath sounds were normal, and no rash was observed. - **Anaphylaxis-**	185% (93.4%)
4) A 13-year-old female patient with a diagnosis of asthma developed shortness of breath 45 min after eating shrimp pasta at a Chinese restaurant with her family. She used 4 puffs of salbutamol inhaler, but 20 min later, widespread itching and persistent shortness of breath led her to visit the emergency department. On physical examination, her general condition was moderate, and she was conscious. Her vital signs were as follows: Temperature: 36.8°C, heart rate: 98 bpm, blood pressure: 110/70 mmHg, respiratory rate: 40 breaths per minute, and oxygen saturation: 90%. Auscultation revealed widespread bilateral rhonchi and wheezing throughout expiration. No rash was observed. - **Anaphylaxis-**	149 (75.3%)
5) A 6-year-old male patient presented to the emergency department after developing hoarseness and difficulty swallowing within 30 min of a bee sting while playing in the park. On physical examination, his general condition was moderate, and he was conscious. His vital signs were as follows: Temperature: 37.2°C, heart rate: 102 bpm, blood pressure: 100/70 mmHg, respiratory rate: 24 breaths per minute, and oxygen saturation: 94%. Auscultation revealed normal breath sounds, and uvular edema was noted. No rash was observed. - **Anaphylaxis-**	145 (73.2%)
6) A 14-year-old male patient with a diagnosis of atopic asthma and pollen allergy developed shortness of breath and wheezing while playing football on a grass field. He administered an additional dose of his regular inhaler containing budesonide + formoterol fumarate, but his respiratory distress did not improve, prompting him to visit the emergency department. On physical examination, his general condition was moderate, and he was conscious. His vital signs were as follows: Temperature: 37°C, heart rate: 102 bpm, blood pressure: 110/65 mmHg, respiratory rate: 38 breaths per minute, and oxygen saturation: 86%. Auscultation revealed widespread bilateral rhonchi throughout expiration, and subcostal retractions were observed. - **Asthma exacerbation-**	147 (74.2%)
7) A 17-year-old female patient, who is being followed for acute rheumatic fever and receiving prophylaxis, presented to the emergency department for a benzathine penicillin injection. Five minutes after receiving the intramuscular injection, she fainted and was transferred to the resuscitation area. On physical examination, her general condition was moderate, and she was confused. She appeared pale, with the following vital signs: Temperature: 37°C, heart rate: 58 bpm, blood pressure: 80/50 mmHg, respiratory rate: 38 breaths per minute, and oxygen saturation: 95%. The ECG was consistent with sinus bradycardia, and auscultation revealed normal breath sounds. - **Vasovagal syncope-**	67 (33.8%)
8) A 7-year-old male patient, who is being followed for severe combined immunodeficiency (SCID), developed a fever of 39°C and a headache during IVIG administration. His vital signs were as follows: Heart rate: 140 bpm, blood pressure: 100/60 mmHg, respiratory rate: 24 breaths per minute, and oxygen saturation: 96%. On physical examination, his general condition was moderate. An ECG showed sinus tachycardia, and breath sounds were normal on auscultation. - **Nonallergic reaction-**	185 (93.4%)
9) A 12-year-old female patient developed facial flushing, a sensation of heat, tingling in her hands, and a headache 1 h after eating mackerel at a fish restaurant with her family. Upon presentation to the emergency department, her vital signs were found to be normal, and her symptoms resolved after administration of an antihistamine. - **Scombroid reaction-**	176 (88.9%)
10) A 2-year-old male patient with a history of anaphylaxis to milk-based formula entered the kitchen while his mother was boiling milk. Fifteen minutes later, he developed wheezing and coughing. Upon presentation to the emergency department, his physical examination revealed a moderate general condition. His vital signs were as follows: Temperature: 37°C, heart rate: 120 bpm, blood pressure: 90/60 mmHg, respiratory rate: 52 breaths per minute, and oxygen saturation: 90%. Auscultation revealed widespread bilateral wheezing. No rash was observed. - **Bronchospasm-**	Bronchospasm: 71 (35.9%) Anaphylaxis 127 (64.1)

There were multiple choice and open-ended questions regarding knowledge on anaphylaxis treatment. Data on the questions and response rates are shown in Table 2. Residents in the last 2 years of their training and those who had previously received anaphylaxis training from pediatric allergy specialists were found to have significantly higher correct response rates on all 16 questions, including knowledge-based items (p < 0.001, p = 0.002).

**Table 2:** Questions on knowledge of treatment of anaphylaxis

Questions	Answers (n,%)
1) What is the first drug that should be administered in the treatment of anaphylaxis?	
Antihistaminic	1 (0.5%)
Methylprednisolone	0 (0%)
Adrenaline	197 (99.5%)
2) Which route should adrenaline be administered in anaphylaxis?	
Intravenous	2 (1%)
Subcutaneous	3 (1.5%)
Intramuscular	193 (97.5%)
3) Where is the recommended injection site for adrenaline?	
Deltoid muscle	11 (5.6%)
Vastus lateralis	177 (89.4%)
Gluteus maximus	8 (4%)
I don't know	2 (1%)
4) What is the recommended dose of adrenaline in the treatment of anaphylaxis?	
Correct	161 (81.3%)
**Have you ever received any training on the principles of using an adrenaline auto-injector?**	
• Yes	179 (90.4%)
**If your answer is yes, who provided the training?**	
• Pediatric allergy and immunology specialist	89 (44.9%)
• Pediatric health and diseases specialist	62 (31.3%)
• Senior resident	28 (14.1%)
**Have you ever provided any training to patients on the principles of using an adrenaline auto-injector?**	
• Yes	51 (25.8%)
**Have you ever prescribed an adrenaline auto-injector?**	
• Yes	60 (30.3%)

## Discussion

In our study conducted to assess the knowledge of pediatric residents regarding when allergic reactions should be considered as anaphylaxis and how they should be treated, we observed a weak consensus among residents on the correct diagnosis and differential diagnosis of anaphylaxis. In only half of the case scenarios were at least 75% of participants in agreement on the appropriate approach.

Liberman et al in a study like ours, designed with case scenarios to assess whether a case represented anaphylaxis and the necessity of administering adrenaline, included 1001 pediatric specialists. Similar to our findings, they observed a lack of consensus on when an allergic reaction should be classified as anaphylaxis and when the administration of adrenaline is appropriate. The authors emphasized the need for education on the diagnosis of anaphylaxis and the use of adrenaline as a first-line treatment.^6^

One of the questions in our study with a low rate of success (66.2%) was the second case scenario. We believe that the main reason for missing the diagnosis of anaphylaxis in this case was the absence of skin/mucosal symptoms. Although studies show that skin involvement in anaphylaxis in children is very common, with a rate as high as 92.7%,^7^ it should be emphasized during training that skin/mucosal involvement is not an essential diagnostic criterion, in order to prevent misdiagnosis of such cases.

Vasovagal syncope (VVS) typically occurs due to fear or pain during injections. VVS stems from abrupt changes in autonomic activity: increased parasympathetic activity (ie, increased vagal tone) leads to bradycardia, and decreased sympathetic activity causes arterial relaxation, resulting in hypotension. If the decrease in cardiac output and blood pressure (BP) is severe, cerebral blood flow may significantly decrease, leading to loss of consciousness. Differentiating between VVS and anaphylaxis may be challenging due to the overlap in some common signs and symptoms, potentially affecting diagnosis and treatment. Key points to consider when distinguishing anaphylaxis from VVS include differences in heart rate (HR), BP, the speed of onset and resolution, the patient's positioning, and the likelihood of exposure to triggering agents.^8^ Responses to the seventh case scenario in our study indicate a low level of awareness (33.8%) regarding VVS as an important differential diagnosis. As in the case scenario, syncope occurring shortly after medication administration is often confused with anaphylaxis. It is important to emphasize that bradycardia and pallor, which occur during these reactions, are distinguishing features that should be highlighted in resident education.

As observed in the case presented in Scenario 10 of our study, lower respiratory symptoms such as bronchospasm triggered by inhalation of food allergens—in the absence of ingestion—do not fulfill the diagnostic criteria for anaphylaxis, according to the 2020 WAO guidelines. This distinction holds significant importance in clinical practice, as respiratory symptoms caused by inhalation may be mistakenly attributed to anaphylaxis, particularly in pediatric patients with a known history of food allergy. Our study contributes to the identification of such conceptual misconceptions by revealing how physicians interpret and respond to these ambiguous clinical scenarios. These findings underscore the need for more comprehensive education on the diagnostic criteria for anaphylaxis within pediatric residency training programs.

In another study involving 173 physicians, where their awareness of anaphylaxis diagnosis and management was evaluated through case scenarios, it was found that while 42.8% of participants knew the first-line treatment for anaphylaxis, only 24.3% and 24.9% correctly answered questions about the appropriate dose and route of adrenaline administration, respectively.^9^ In a survey study conducted in Egypt, 40.5% of 242 specialist physicians considered adrenaline as the first-line treatment, but only 31% knew the correct dose, and 49.2% knew the correct route of administration.^10^ In another study conducted in Turkey among pediatric specialists, adrenaline was preferred as the first-line treatment for anaphylaxis by 99.2% of participants, with 82.8% correctly answering the dose, 88.9% the route, and 89.7% the site of administration.^4^ Similarly, in our study, adrenaline was preferred as the first-line treatment for anaphylaxis in 99.5% of cases, with 81.3% correctly answering the dose, 97.5% the route, and 89.4% the site of administration. The higher rates in our study compared to adult studies were attributed to the fact that our center is a reference hospital that also accepts emergency patients, and that anaphylaxis training is periodically repeated.

It was emphasized that supporting theoretical lessons on anaphylaxis diagnosis and treatment with practical application training could improve success rates.^11^

It is important to emphasize that administering epinephrine in the acute setting remains safe and appropriate when anaphylaxis is strongly suspected, even if the diagnosis is later ruled out. All patients treated with epinephrine for suspected anaphylaxis should be referred to an allergist for further evaluation. In cases such as vasovagal syncope (Scenario 7) or inhalation-triggered wheeze (Scenarios 6 and 10), epinephrine may be administered based on clinical suspicion. Similarly, a patient presenting with acute urticaria and vomiting may receive epinephrine, only to later be diagnosed with acute spontaneous urticaria associated with gastroenteritis or somatization during a panic attack. However, the resolution of symptoms following epinephrine administration does not necessarily indicate that the symptoms were due to anaphylaxis or of allergic origin.

In conclusion, although the understanding of anaphylaxis has come a long way in the 120 years since it was first described, ambiguity in its definition remains. Lack of accurate, rapid, and objective tests makes the diagnosis difficult, contributing to the burden on both the care providers managing acute episodes and those evaluating the patient for etiologies after the event. Various approaches, such as organizing in-service training programs on anaphylaxis diagnosis and emergency treatment, repeating these trainings periodically, and utilizing reminder posters, can help reduce knowledge gaps among physicians and enhance their clinical skills. It should also be emphasized during training sessions that some cases of anaphylaxis can occur without any cutaneous manifestations.

## Abbreviations

NIAID, The National Institute of Allergy and Infectious Diseases; FAAN, Food Allergy and Anaphylaxis Network; WAO, World Allergy Organization; EAACI, European Academy of Allergy and Clinical Immunology; IQR, interquartile range; VVS, Vasovagal syncope; BP, Blood pressure; HR, Heart rate.

## Author contributions

**Tülay Tuğçe Kutsal Gültekin**: Data curation (equal), formal analysis (equal), investigation (equal), methodology (equal), resources (equal), writing-original draft (equal), writing-review and editing (equal); **Ayşe Gökçe Kutsal**: Data curation (equal), formal analysis (equal), resources (equal); **Gökhan Yörüsün:** Data curation (equal), formal analysis (equal), resources (equal); **Ahmet Selmanoğlu:** Data curation (equal), formal analysis (equal), resources (equal); **Kaan Çelebier:** Data curation (equal), formal analysis (equal), resources (equal); **Zeynep Şengül Emeksiz:** Data curation (equal), formal analysis (equal) resources (equal); **Emine Dibek Mısırlıoğlu:** Project administration (equal), data curation (equal), methodology (equal), formal analysis (equal) writing-original draft (equal), writing-review and editing (equal).

## Ethics

The approval of the Ethics Committee for Clinical Studies (Decision No: TABED 2-24-111) was obtained for our study, and a written informed consent form was obtained from all participants before inclusion in the study (approval date: April 03, 2024).

## Authors’ consent for publication

All authors approved the manuscript and gave their consent for submission and publication.

## Availability of Data and Materials

Data and materials are not available.

## Funding/support

The study did not receive funding.

## Declaration of competing interest

The authors have no conflicts of interest to declare.
